# Marginal role for 53 common genetic variants in cardiovascular disease prediction

**DOI:** 10.1136/heartjnl-2016-309298

**Published:** 2016-06-30

**Authors:** Richard W Morris, Jackie A Cooper, Tina Shah, Andrew Wong, Fotios Drenos, Jorgen Engmann, Stela McLachlan, Barbara Jefferis, Caroline Dale, Rebecca Hardy, Diana Kuh, Yoav Ben-Shlomo, S Goya Wannamethee, Peter H Whincup, Juan-Pablo Casas, Mika Kivimaki, Meena Kumari, Philippa J Talmud, Jacqueline F Price, Frank Dudbridge, Aroon D Hingorani, Steve E Humphries

**Affiliations:** 1 School of Social & Community Medicine, University of Bristol, Bristol, UK; 2 Department of Primary Care & Population Health, University College London, London, UK; 3 Centre for Cardiovascular Genetics, Institute of Cardiovascular Science, University College London, London, UK; 4 Institute of Cardiovascular Science and Farr Institute, University College London, London, UK; 5 MRC Unit for Lifelong Health and Ageing at UCL, London, UK; 6 MRC Integrative Epidemiology Unit, School of Social and Community Medicine, University of Bristol, Bristol, UK; 7 Centre for Population Health Sciences, University of Edinburgh, Edinburgh, UK; 8 Department of Non-communicable Disease Epidemiology, London School of Hygiene and Tropical Medicine, London, UK; 9 Division of Population Health Sciences and Education, St George's, University of London, London, UK; 10 Department of Epidemiology & Public Health, UCL Institute of Epidemiology & Health Care, University College London, London, UK; 11 Institute for Social and Economic Research, University of Essex, Colchester, UK

## Abstract

**Objective:**

We investigated discrimination and calibration of cardiovascular disease (CVD) risk scores when genotypic was added to phenotypic information. The potential of genetic information for those at intermediate risk by a phenotype-based risk score was assessed.

**Methods:**

Data were from seven prospective studies including 11 851 individuals initially free of CVD or diabetes, with 1444 incident CVD events over 10 years' follow-up. We calculated a score from 53 CVD-related single nucleotide polymorphisms and an established CVD risk equation ‘QRISK-2’ comprising phenotypic measures. The area under the receiver operating characteristic curve (AUROC), detection rate for given false-positive rate (FPR) and net reclassification improvement (NRI) index were estimated for gene scores alone and in addition to the QRISK-2 CVD risk score. We also evaluated use of genetic information only for those at intermediate risk according to QRISK-2.

**Results:**

The AUROC was 0.635 for QRISK-2 alone and 0.623 with addition of the gene score. The detection rate for 5% FPR improved from 11.9% to 12.0% when the gene score was added. For a 10-year CVD risk cut-off point of 10%, the NRI was 0.25% when the gene score was added to QRISK-2. Applying the genetic risk score only to those with QRISK-2 risk of 10%–<20% and prescribing statins where risk exceeded 20% suggested that genetic information could prevent one additional event for every 462 people screened.

**Conclusion:**

The gene score produced minimal incremental population-wide utility over phenotypic risk prediction of CVD. Tailored prediction using genetic information for those at intermediate risk may have clinical utility.

## Introduction

Despite the importance of predicting future cardiovascular disease (CVD) among initially healthy adults, predictive accuracy has often seemed disappointing, as most individuals who eventually suffer a CVD event were previously at average risk rather than high risk: the prevention paradox.^1^ Lowering cholesterol through statin use reduces CVD risk.^2^ Accordingly, several major guidelines^3–6^ recommend lipid-lowering therapy for people with a raised 10-year CVD predicted risk, traditionally using a threshold of 20%. However, with recent patent expiries resulting in reduced acquisition cost, and increasing evidence on the limited harms of statins, the 10-year CVD risk threshold for primary prevention of CVD has been reduced to 10% in the UK^3^ and to 7.5% in the USA.^4^ However, these decisions have been questioned, especially since people with intermediate 10-year CVD risk (eg, 10%–20%) may be reluctant to undergo statin therapy.^5^ Refining risk estimation may be of particular interest in such individuals, as well as helping guide appropriate targeting of alternative therapies currently under development.

Considerable advances have taken place in understanding genetic determinants of CVD in recent years and the CardiogramPlusC4D collaboration have now catalogued associations of hundreds of thousands of single nucleotide polymorphisms (SNPs) across the genome, using data on over 63 000 coronary heart disease (CHD) cases and 130 000 controls.^6^ This collaboration identified 46 loci containing SNPs that surpassed genome-wide levels of statistical significance. Further SNPs associated with ischaemic stroke risk have included rs783396 from the *AIM1* gene in chromosome 6q21^7^ and rs12425791 (closest gene *NINJ2*, chromosome 12).^8^ Case-control studies do not permit estimation of absolute risk. We, therefore, evaluated the predictive performance of a gene score based on 53 SNPs associated with CHD or stroke on its own and in conjunction with the established non-genetic QRISK-2 risk tool^9^ (developed for CVD prediction in UK populations), using the University College-London School-Edinburgh-Bristol (UCLEB) Consortium of prospective population studies.^10^

## Methods

### UCLEB Consortium

A full description of the UCLEB Consortium has been previously published.^10^ Briefly, the studies comprise individuals almost exclusively of European ancestry from a wide geographical range within the UK. For the current analysis, seven prospective studies with genotype and complete information on CVD incidence were included. For full details of individual studies, see online supplementary information. In four of the studies (Edinburgh Artery Study (EAS), MRC National Study of Health and Development (NSHD), Whitehall II study (WHII) and Caerphilly Prospective study (CaPS)), all participants providing blood samples were genotyped, but a nested case-control sample was used for the remainder. Analysis was restricted to 11 851 individuals aged ≤85 years and excluded 1542 individuals with prevalent diabetes and 1191 with prevalent CVD.

10.1136/heartjnl-2016-309298.supp1 Supplementary data

Informed consent was obtained for all subjects included in UCLEB research. Written approval from individual Research Ethics Committees to use anonymised individual-level data has been obtained by each participating study.

### Clinical characteristics of the participants

Within individual cohorts, biochemical measurements were performed in accredited laboratories using international standards.^10^ For the current analysis, earliest available measurements were abstracted for each study on relevant phenotypes. Medication data included lipid-lowering drugs (statins or other) and blood pressure-lowering drugs; for the latter, adjustment was made by adding 15 mm Hg for systolic and 10 mm Hg for diastolic blood pressure.^11^

### Definition of CVD

The definition of prevalent CVD (from the same time point as the phenotypic measurements) was based on either self-report, medical record review or examination with ECG. CVD consisted of a combination of CHD and stroke. CHD included all non-fatal myocardial infarction or any revascularisation procedure (coronary artery bypass surgery or angioplasty) and fatal CHD. Stroke included all non-fatal stroke (ischaemic and haemorrhagic combined, but excluding transient ischaemic attacks) and fatal stroke. Fatal events were classed according to International Classification of Diseases-10 codes: I20–I25 for CHD and I60–I69 for stroke.

### Genotyping

DNA was extracted from blood samples either collected at baseline (British Women's Heart and Health Study (BWHHS)) or at a subsequent resurvey (British Regional Heart Study (BRHS), MRC NSHD, EAS, WHII, English Longitudinal Study of Ageing (ELSA), CaPS).^10^ Genotype data were based on the Illumina CardioMetabochip, which incorporates approximately 200 000 SNPs from loci previously identified for associations with cardiometabolic disease risk factors and outcomes.^12^ Imputation was conducted against the 1000 genomes reference panel, providing information on approximately 2 million typed or imputed SNPs. Duplicate samples were genotyped to compute the error rate. Quality control on genotyped samples has been previously reported^10^ and all included SNPs had a call rate of >98%. Genotypes were in Hardy Weinberg Equilibrium in all studies.

We used the list of CVD-risk SNPs recently identified in large meta-analyses of CHD^6^ and stroke^7^ ^8^ (see online supplementary file, eTable 1); all 53 CVD SNPs except one were typed through the CardioMetabochip: one SNP associated with stroke (rs783396) was imputed.

### Statistical analysis

#### Score construction

We used the QRISK-2 2014 batch processor, using data for age, sex, smoking, family history of CVD, body mass index, blood pressure, treatment for hypertension, total and high-density lipoprotein (HDL)-cholesterol, to compute the QRISK-2 risk probabilities.^9^ We computed a genetic risk score (GRS) weighted according to published coefficients (log ORs) for the 53 SNPs.^6^ Coefficients were multiplied by 0, 1 or 2, according to the number of risk alleles carried by each person. The logits of the QRISK-2 probabilities were added to the GRS to produce a combined score. As a sensitivity analysis, to address concerns that β-coefficients for the individual SNPs selected for the GRS may be inflated, we calculated an unweighted gene score and followed similar procedures.

#### Association testing

Logistic regression models were fitted to obtain the OR per SD increase in the GRS as well as OR associated with each quintile. Association models were fitted using the combined dataset with a term for study included as a fixed effect.

#### Model discrimination

We calculated the area under the receiver operating characteristic curve (AUROC) and the detection rate, defined as the proportion of all cases detected for a false-positive rate (FPR) of 5% (DR5) and 10% (DR10). AUROCs were calculated separately for each study and combined using both fixed effects and random effects meta-analysis. Improvements in discrimination were assessed by calculating the difference between the two AUROCs in each study with bootstrap estimates of the CI and then combining these over the studies.

#### Model calibration

For the combined score, estimates of risk were obtained by converting the logit back to a probability. For all studies but ELSA, the number of events occurring within 10 years of baseline was observed. For ELSA, since follow-up was for 5 years only, we doubled this to give the 10-year observed risk. Observed risks were then compared with predicted risks within tenths of the predicted risk distribution and the Hosmer-Lemeshow test was used to assess goodness of fit.

#### Reclassification of CVD risk

We used the net reclassification improvement (NRI) index to evaluate improvement in risk prediction. This metric quantifies the extent to which the combined score moved people to risk categories that better reflected their future event status.^13^ In three of the studies, all cases were genotyped but only a fraction of the controls so it was necessary to upweight data for controls to reflect properly the proportion of cases in the population. For example, if within a particular age group of one study, only 80% of controls had been selected for genotyping, we assigned a weight of 1.25 (=100/80) to all those controls but a weight of 1 to cases, when calculating the number who had been reclassified. We used three 10-year CVD risk categories (<10%, 10%–19.9% and 20% or higher). We calculated the NRI without accounting for study and then calculated NRI and its standard error for each study and combined it to an overall NRI with a fixed-effects meta-analysis. As there was very little difference in the two methods, we present results for the latter.

We also followed the Emerging Risk Factors Collaboration's method^14^ in assessing additional predictive value of novel risk factors for individuals initially categorised as intermediate risk according to established risk factors. Of those whose predicted risk was between 10% and 20% according to the QRISK-2 equation, we calculated the number who would subsequently be reclassified as high risk once the GRS was added. We assumed all such individuals would be treated with statins and would achieve a 20% relative risk reduction (adherence assumed to be similar to that seen in trials^2^) and from this we estimated the absolute number of cardiovascular events that might be prevented. This enabled us to calculate the number needed to screen to prevent one event.

All analysis was conducted using Stata (V.13.1; StataCorp, Texas, USA).

## Results

### Characteristics of the study participants

**Figure 1:**
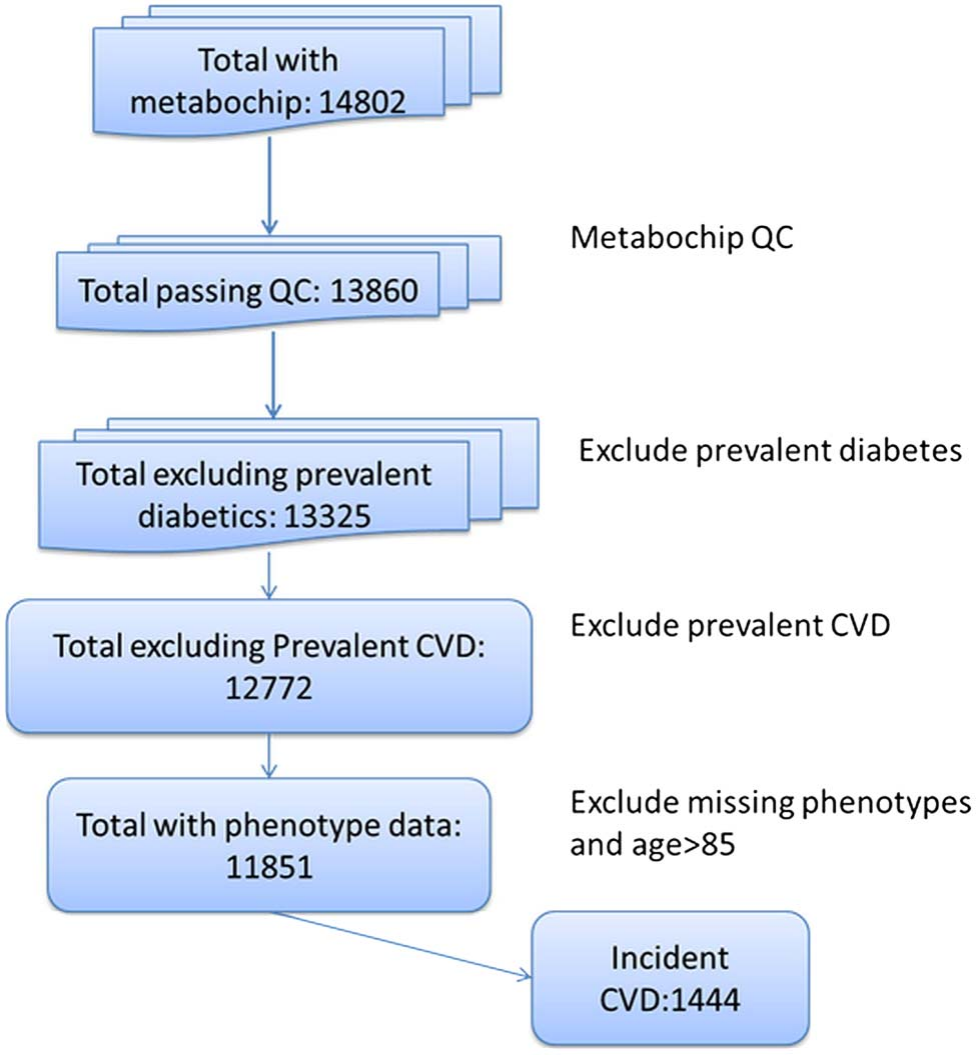
Flow chart showing the selection of participants for analysis. CVD, cardiovascular disease.

Studies differed by sex and age (table 1). A total of 1444 individuals out of 11 851 (1054 CHD events, 390 strokes) experienced CVD within 10 years of follow-up (figure 1). A total of 297 events were fatal. The 10-year CVD event rates varied by study, from 4.7% in NSHD (mean age 53 years at baseline of follow-up) to 37.2% in EAS (mean age 64.2 years). Only 165 of the participants (1.4%) were on statin treatment at the start of follow-up.

**Table 1:** Characteristics of participants in the seven studies

	BRHS	BWHHS	CaPS	EAS	ELSA	NSHD	WHII	Total
Total	2138	1631	1121	632	1184	2330	2815	11 851
Follow-up (years)	10	10	10	10	∼5	10	10	
CVD events during follow-up (n)	205	268	119	235	142	109	366	1444
10-year CVD event rate (per 100 person-years)	8.4*	11.4*	14.7	37.2	20.8*†	4.7	15.2	13.6
Predicted 10-year CVD risk (QRISK-2) (per 100 person-years)	8.4	26.7	18.1	20.6	30.2	10.7	8.2	15.1
Age (years)	48.9 (5.6)	70.7 (5.3)	56.7 (4.4)	64.2 (5.7)	71.5 (8.5)	53.0 (0.0)	48.8 (6.0)	
Sex, % male	100	0	100	46.4	51.9	49.4	77.2	
Ever smokers (%)	33.1	46.0	80.5	22.6	64.1	72.5	48.3	
Family history of CVD (%)	–	58.2	–	67.6	5.3	63.0	51.6	
Townsend score		0.355 (3.21)					−0.21 (0.91)	
Body mass index (kg/m^2^)	25.4 (2.9)	27.4 (4.8)	26.5 (3.6)	25.2 (3.6)	27.3 (4.2)	27.2 (4.5)	25.1 (3.5)	
Total cholesterol (mmol/L)	6.36 (1.04)	6.79 (1.21)	5.61 (0.98)	7.08 (1.32)	6.12 (1.21)	6.12 (1.05)	6.44 (1.16)	
Systolic blood pressure (mm Hg)	144.1 (20.2)	154.0 (27.0)	146.0 (22.3)	144.6 (25.1)	144.6 (19.6)	137.7 (21.1)	121.3 (14.0)	
Treated hypertension (%)	2.2	28.6	–	16.8	36.0	11.6	5.8	
Calendar years for baseline data collection	1978–1980	1999–2001	1984–1988	1987–1988	2004–2005	1999	1992–1993	

Mean (SD) tabulated for continuous variables, percentage for binary variables.

*Adjusted for nested case-control study design, accounting for sampling fraction of controls.

†In ELSA, follow-up was for 5 years so the observed number of events was doubled for the 10-year rate.

CVD, cardiovascular disease.

### GRS and association with CVD risk factors and CVD events

Not every SNP demonstrated similar associations with CVD in the UCLEB data to those previously published (see online supplementary file, eTable 1), with ORs <1 for 14 of the 53 SNPs in the UCLEB data.

There was a clear positive relationship of the GRS with total cholesterol and an inverse relationship with HDL cholesterol (see online supplementary file, eTable 2). These associations attenuated when eight SNPs related to low-density lipoprotein concentration were excluded from the gene score. Only a very modest positive association was seen with reported family history.

ORs of incident CVD for successive quintiles of the GRS compared with the lowest quintile were 0.88, 1.10, 1.12 and 1.15, respectively, with an OR of 1.09 per SD increase (95% CI 1.03 to 1.15, p=0.005). Restricting incident CVD cases to 137 fatal events within 10 years, the OR for the GRS per SD increase was 1.03 (95% CI 0.87 to 1.22, p=0.74). When considering prevalent CVD cases, the equivalent OR was 1.17 (95% CI 1.10 to 1.25, p=8.2×10^−7^). The relationship of the QRISK-2 score with all incident CVD events was much stronger (OR per SD increase 1.92: 95% CI 1.78 to 2.08, p=2.6×10^−58^).

### Predictive accuracy of the GRS alone and in combination with QRISK-2

Table 2 shows the AUROCs, for the GRS (0.524) and QRISK (0.635) alone and the two in combination (0.623; see also online supplementary file, eTable 3), as well as the detection rates for 5% and 10% FPRs. These AUROC estimates were virtually identical when family history data were not used for the QRISK-2 score and also when random effects instead of fixed-effects analysis was used to combine studies' results. Detection rates for 5% and 10% FPRs were 6.8% and 13.1%, respectively, for the GRS alone. The corresponding detection rates for QRISK-2 were 11.9% and 21.2%, changing to 12.0% and 19.6%, respectively, when the GRS was added.

**Table 2:** Area under the receiver operating characteristic curve (AUROC) (95% CI) and detection rates for the combined data

	AUROC for combined studies	Detection rate for 5% false-positive	Detection rate for 10% false-positive
Externally weighted gene score	0.524 (0.508 to 0.541)	6.8% (5.5 to 8.1)	13.1% (11.3 to 14.8)
QRISK-2	0.635 (0.619 to 0.650)	11.9% (10.3 to 13.6)	21.2% (19.1 to 23.3)
QRISK-2+ Externally weighted gene score	0.623 (0.608 to 0.639) p=0.06*	12.0% (10.3 to 13.6)	19.6% (17.5 to 21.6)

*p Value derived from the comparison with QRISK-2 alone, estimated difference (95% CI)=−0.008 (−0.017 to 0.000).

**Figure 2:**
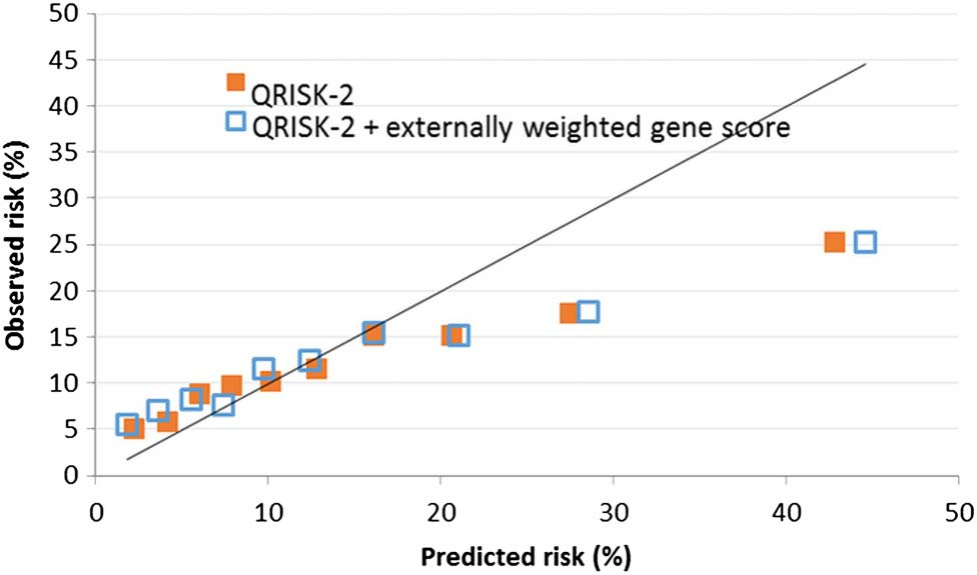
Calibration shown by plot of observed and predicted probabilities of cardiovascular disease within 10 years when predicted risk distribution was divided into tenths. Results are shown for QRISK-2 prediction score and QRISK-2 combined with genetic risk score.

Figure 2 shows that although QRISK-2 was well calibrated with observed risk over the majority of the risk distribution, it modestly underpredicted at low levels of risk and substantially overpredicted risk for those in the top three tenths of the predicted risk distribution. Adding information from the GRS had little effect on calibration: both predictive scores departed significantly from being well calibrated (χ ^2^_8_=309.0 for QRISK-2 and 427.1 for QRISK-2+GRS by the Hosmer-Lemeshow test).

### Reclassification

NRI indices are shown in tables 3 and 4 according to whether individuals were above or below 10% predicted risk (table 3) and whether individuals were above or below 20% predicted risk (table 4). For those who did not actually experience an event, extra 2.33% individuals crossed the threshold downwards rather than upwards when the GRS was added to the QRISK-2 equation. For those who did experience an event, extra 2.08% individuals crossed the threshold downwards rather than upwards when the GRS was added. Overall, the NRI was therefore 0.25% (95% CI −1.33% to 1.83%). When a threshold of 20% was used, a net increase of 0.90% was observed for those who crossed the threshold downwards rather than upwards among those who did not experience events and also a net increase of 0.25% in the same direction for those who did experience events. Hence the NRI was 0.65%.

**Table 3:** Net reclassification index (NRI) based on addition of gene score to QRISK, calculated using 10% risk cut-off

	No. of people				
	QRISK+externally weighted gene score NO CVD (n=15928.64*)		Reclassified		
Predicted risk QRISK	<10	≥10	Increased risk	Decreased risk	Net correctly reclassified
<10 ≥10	5475.02 1156.07	785.56 8511.99	785.56	1156.07	2.33% (1.8 to 2.9)
	QRISK+externally weighted gene score CVD (N=1697.81*)				
**Predicted risk QRISK**	**<10**	**≥10**			
<10 ≥10	352.27 100.58	65.36 1179.60	65.36	100.58	−2.07 (−3.56 to −0.59)
NRI (95% CI)† NRI (95% CI)‡	0.25% (−1.33 to 1.83) p=0.76 1.18% (−0.23 to 2.60) p=0.10				

*Numbers inflated due to extra weighting assigned to three studies where samples of controls were taken (see statistical analysis section).

†No adjustment for study.

‡Results from meta-analysis of individual study results (fixed effects).

CVD, cardiovascular disease.

**Table 4:** Net reclassification index (NRI) based on addition of gene score to QRISK, calculated using 20% risk cut-off

	No. of people				
	QRISK+externally weighted gene score NO CVD (N=15928.64*)		Reclassified		
Predicted risk QRISK	<20	≥20	Increased risk	Decreased risk	Net correctly reclassified
<20 ≥20	9789.24 927.36	783.85 4428.19	783.85	927.36	0.90% (0.39 to 1.41)
	QRISK+externally weighted gene score CVD (N=1707.7*)				
**Predicted risk QRISK**	**<20**	**≥20**			
<20 ≥20	605.5 124.3	146.1 831.9	146.1	124.3	−0.25% (−2.09 to 1.58)
NRI (95% CI)† NRI (95% CI)‡	0.65% (−1.26 to 2.55) p=0.51 0.68% (−1.16 to 2.52) p=0.47				

*Numbers inflated due to extra weighting assigned to three studies where samples of controls were taken (see statistical analysis section).

†No adjustment for study.

‡Results from meta-analysis of individual study results (fixed effects).

CVD, cardiovascular disease.

### Estimated performance of a sequential screening strategy

**Figure 3:**
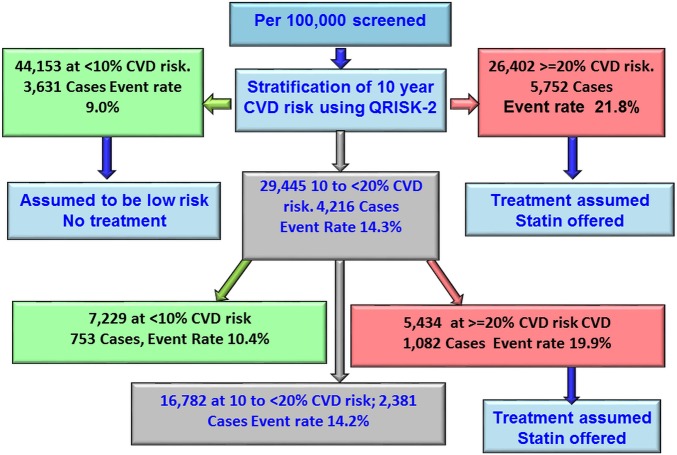
Flow chart showing the modelling of reclassification using Gene Score. CVD, cardiovascular disease.

Figure 3 illustrates the estimated effect of a sequential screening strategy applied to 100 000 people screened for CVD risk using QRISK-2 followed by addition of information from a GRS among those estimated to be in the intermediate-risk category (10 year risk 10% to <20%). Based on QRISK-2 estimates, for every 100 000 people in the population from which our data were drawn, 29 445 would be at intermediate risk. When adding the GRS, 16 782 would remain as intermediate risk, 7229 would be reclassified as low risk and 5434 would be reclassified as high risk, thus making them eligible for statin treatment. Based on extrapolation from the current analysis of those reclassified by addition of the GRS, an estimated 1082 would go on to suffer a CVD event within 10 years. Assuming a 20% reduction in events from statin treatment, 216 events (20% of 1082) would be expected to be prevented. Therefore, adding information from the GRS to QRISK-2 among those classified as being at intermediate risk by the latter would postpone one event for every 462 screened.

### Potential influence of age on screening performance

Discrimination and reclassification was estimated separately for participants aged under 60 and over 60 (see online supplementary file, eTables 4–6). There was no evidence of any differences in AUROC for the GRS alone (0.530 and 0.518, respectively), in the improvement in AUROC of GRS compared with QRISK-2 alone (−0.010 and −0.007, respectively), NRI based on the 10% cut-off point for predicted risk (0.60% for each age group) or NRI based on the 20% cut-point (1.0% and 1.5%).

## Discussion

Our study suggests that gene scores from 53 SNPs were not effective in predicting 10-year risk of CVD, with an area under the curve of only 0.524; this area was 0.635 for a model with QRISK-2 alone and 0.623 when a GRS was added in the model. Nevertheless, the GRS appeared to carry some utility when applied only to those who, according to conventional risk scoring, would have been classified at intermediate risk, by moving some individuals into the high-risk category. Among 100 000 people from a population represented by our combined studies, 29 445 would be classed as of intermediate risk according to the QRISK equation, but of these, 5434 would then be reclassified as high risk once the GRS was applied and 1082 would suffer a CVD event if untreated with statins. For 20% of these (216 people), treatment with statins according to guidelines could postpone the CVD event, leaving a number needed to screen of 462 to postpone one CVD event. Recent evidence suggests that the risk reductions from statin therapy might be enhanced for those at highest risk,^15^ so this figure may be conservative.

Our data include seven British prospective studies, in which CVD events were defined in a standard manner,^10^ genotyping followed a common protocol and almost 1500 incident CVD events were available for analysis. The participants of the studies were a median of 53 years and more commonly male. This age group represents a population group most eligible for cardiovascular screening and we did not see differential performance of the screening algorithms according to age group, even when we restricted analysis to those aged ≤53 (data not shown). Genetic information may be more useful for those aged <40 (not represented in this study), but a lifetime risk equation would then be required. In all seven studies combined, we noticed substantial overprediction by QRISK-2, despite its apparently good calibration in other UK-based prospective studies.^16^ Thus, while we noted that a two-stage screening procedure would yield identification and treatment of some high-risk individuals who would have been classified at intermediate risk by QRISK-2, the phenomenon of overprediction by QRISK-2 suggests that many more needed to be reclassified as low risk. The genetic score did not actually improve the calibration at all.

In constructing the GRS, we used regression coefficients catalogued by the CardiogramplusC4D consortium on a very large dataset. While the regression coefficients for SNPs extracted from this dataset will perform less optimally when applied to a new dataset, we believe this represents a truer test of validation.^17^ The 53 SNPs will probably be those SNPs most strongly associated with CVD that will ever be found, but their combined effect still represents a small proportion of heritability of CVD and is still small compared with major phenotypic risk factors. Better prediction from genotypic information may be expected from identification of several thousand more SNPs.^18^

The development of QRISK-2, and most of our studies' baselines, pre-dated the statin era and the proportion taking statins during follow-up would be modest. Our data are capable of evaluating what risks could have been prevented had statins been widely available.

Other attempts to evaluate use of genotypic data for cardiovascular risk screening have been made. A marginal improvement in discrimination over and above the predictive power of traditional coronary risk factors was found in the ARIC study for African-American participants (but less clearly for Caucasian participants)^19^ and among European men.^20^ Among participants of the Framingham study, no significant improvement in discrimination was found but a modest benefit in reclassification of CVD risk.^21^ The Framingham study and the REGICOR study (north-eastern Spain, low CHD risk) were used to assess CHD risk: this showed that a GRS improved discrimination for Framingham participants but not REGICOR.^22^ However, better performance was seen for reclassification of those at intermediate risk in both studies. The same was true in the FINRISK studies,^23^ which estimated with a two-stage screening that 135 events could be prevented among 100 000 screened, slightly less than 216/100 000 in the present study.

Recent data from the Malmὂ Diet and Cancer Study showed that family history did not lessen the predictive utility of a GRS, but the GRS added predictive value over phenotypic risk scores which included family history.^24^ In contrast, our data find little evidence for improvement in discrimination over a phenotypic risk score, whether or not it includes family history.

The Rotterdam study^25^ conducted similar GRS analysis using the same subset of 53 SNPs as in the present study. As in our study, a stronger relationship of gene score was observed for prevalent cases than incident. The present study also observed a weaker relationship of gene score with CVD mortality, thereby supporting the suggestion that some genes identified by CardiogramplusC4D were related to better survival after CVD, rather than to incident disease, and questions the generation of signals through genome-wide association studies in case-control studies, if no distinction can be made between cases who have died and those who survived. A fully powered prospective study is required of individuals with incident CVD, to compare genotypes between survivors and those who died of the event.

Our findings underline the relatively disappointing performance of gene scores in adding to cardiovascular risk scores based on established risk factors. Nevertheless, we have shown the potential for refining risk calculation in those initially classed as of intermediate risk. A similar analysis applied to selective use of C reactive protein and fibrinogen in those at intermediate risk suggested that these markers would require over 3000 screened to postpone a CVD event:^14^ the relatively better performance of the GRS in the present study is because a higher proportion of those at intermediate risk were reclassified as high risk. It has been shown that a collection of alternative risk scores (including QRISK-2), based on established risk factors, are liable to disagree over classifying individuals as high risk.^26^ Therefore including a GRS may help identify an intermediate group who should properly be classed as of high risk. Despite current UK recommendations that treatment with statins be extended to those at intermediate risk^3^ (CVD risk 10–20% over 10 years) as well as those at high risk (over 20%), family physicians may be reluctant to do so. The Joint British Societies' (JBS3) consensus recommendations for the prevention of CVD did not recommend the use of genetic information, which was seen as currently performing less well than established risk factors.^27^ However for individuals not meeting the criteria for lifestyle or drug therapy, JBS3 recommended calculation of metrics such as heart age, relating to lifetime risk. A gene score with good predictive power would seem particularly suitable to evaluate lifetime risk, given its non-modifiable nature throughout the life course.

The Rotterdam study^25^ constructed a second risk score based on 169 SNPs including the original 53 modelled in our study, as well as a further 116 for whom only modestly significant changes in risks were demonstrated. This second risk score performed better than the first and further gene discovery may therefore produce greater improvements. However, at present, our results and those of others cannot support the population-wide use of GRSs in targeting treatment, despite the modest utility in reclassifying those at intermediate risk.

**Key messages**

**What is already known on this subject?**

- Predictive accuracy of cardiovascular risk, generally based on well-established phenotypic measures, has often seemed disappointing. Genome-wide association studies have highlighted new genetic loci related to coronary artery disease and stroke.

**What might this study add?**

- When information on 53 single nucleotide polymorphisms about individuals from seven UK prospective studies are added to a well-established cardiovascular risk score, the ability to predict cardiovascular disease (CVD) over the next 10 years is not enhanced.
- However, if a genetic risk score is applied to individuals classed at intermediate risk according to a traditional risk score, some individuals will be reclassified at high risk and CVD events will be postponed due to timely use of lipid-lowering therapy. This two-stage strategy will postpone 216 events in every 100 000 people screened.

**How might this impact on clinical practice?**

- Routine use of genetic profiles is not necessary for everyone screened for cardiovascular risk. However, there may be clinical utility for a genetic risk score for those initially screened as of intermediate risk.
