# Supplementary material for: Marginal role for 53 common genetic variants in cardiovascular disease prediction
Source: Heart. 2016 Jun 30;102(20):1640–7. doi: 10.1136/heartjnl-2016-309298 (PMC5099215; doi:10.1136/heartjnl-2016-309298)
Supplement: Supplementary data [file heartjnl-2016-309298supp.pdf]

Supplementary Research Design

Details of the Prospective cohorts used in this analysis

A full description of the UCLEB Consortium has been previously published[1]. The 12 UCLEB studies comprise individuals almost exclusively of European ancestry and cover a wide geographic range within the UK. Population structure was assessed by principal components analysis (PCA) and outliers were excluded. Participants were all at least 40 years old at baseline.

British Regional Heart Study (BRHS): (1) From 1978 to 1980, 7735 men aged 40-59 were recruited from general practices across the UK. A wide range of phenotypic measures is available for established risk markers such as lipids, blood pressure and inflammatory and haemostatic markers. Most of these measures were taken both at recruitment and re-examination, which occurred in 1998-2000 when men were aged 60-79. At this re-examination 4252 participants attended and DNA was extracted for 3945. A case-control sample was selected using 1095 cases with prevalent data at re-examination or incident cases of CHD or stroke over the next 8 years and 1358 controls. The controls were frequency matched based on being in the same town and within the same fixed 5-year age band as the cases. Data on important behavioural variables such as cigarette and alcohol consumption, as well as physical activity, have been regularly collected through follow up. Well validated outcome variables including major coronary heart disease, stroke, diabetes, and revascularisation, as well as cause-specific mortality, continue to be collected from medical records almost 30 years after recruitment. (http://www.ucl.ac.uk/pcph/research-groups-themes/brhs-pub)

The British Women's Heart & Health Study (BWHHS) is a prospective cohort study of 4,286 women aged between 60 and 79 at baseline in 1999-2001. Participants were randomly selected from general practice registers in 23 towns across England, Wales and Scotland. The criteria for the sampling frame and clinic protocols were very similar to the 20 year follow-up of BRHS. Baseline measurements included biomarkers and blood samples for DNA extraction taken by research nurses as well as ascertainment of a wide range of phenotypic measures. Follow-up by postal questionnaire was undertaken in 2003, 2007 and 2010-2011. Of the 4,278 participants who gave consent for genetic studies, 15 were defined by the examining nurse as being nonwhite and were excluded from further analysis. Of the remaining 4,263 women, 3,800 (89%) had DNA available for genotyping. Survival status is obtained from the Data Linkage Service, Health and Social Care Information Centre, London and CVD events have been prospectively studied by biennial review of primary care medical records with validation checks. The UCLEB case-control sample was selected based on 523 cases with prevalent or incident CHD or stroke. 1501 controls were frequency matched based on being in the same town and within the same fixed 5-year age band as the cases. For these analyses prevalent T2D was ascertained from either self-report, medical record review, use of glucose lowering medication, and/or a fasting glucose >7mmol/L. Ethical approval was granted for the BWHHS from the London Multi-Centre Research Ethics Committee and 23 Local Research Ethics Committees.

Medical Research Council National Survey of Health and Development (MRC NSHD): (3) This is an on-going prospective birth cohort study consisting of all births in England, Scotland and Wales in one week in March 1946. The sample includes single, legitimate births whose fathers were in non-manual or agricultural occupations and a randomly selected one in four of all others, whose fathers were in manual labour. The original cohort comprised 2,547 women and 2,815 men who have been followed-up over 20 times since their birth. The data collected to date include repeat cognitive function, physical, lifestyle and anthropomorphic measures, as well as blood analytes and other measures. The cohort recently completed a particularly intensive phase of clinical assessment and biological sampling with blood and urine sampling and analysis, and cardiac and vascular imaging (4).

DNA was extracted from blood samples collected in 1999 (5). Follow-up for disease outcomes is by self-reports of doctor diagnosed events that have been validated against General Practice (GP) records. (http://www.nshd.mrc.ac.uk/).

Edinburgh Artery Study (EAS): (6) At baseline (August 1987-September 1988), an age-stratified random sample of men and women, aged 55-74 years, was selected from the age-sex registers of ten general practices with catchment populations spread geographically and socioeconomically throughout the city of Edinburgh. Subjects were excluded if they were unfit to participate (e.g. due to severe mental illness or terminal disease). These exclusions were replaced by other randomly sampled subjects. The study population is almost exclusively European. DNA was extracted at 5 years follow-up. Physical examinations were performed by specially trained research nurses using standardised operating procedures. The quality of clinical measurements were checked before and during the study by repeat measurements taken intermittently by the study co-ordinator. Individual observer measurements were assessed for drift. Blood assays were performed in accredited laboratories using international standards. Subjects have been followed up for 20 years for cardiovascular events, using repeat self-reporting questionnaires, record linkage for hospitalisations and deaths, and validation of events against prespecified criteria through searching of hospital and GP notes. Comprehensive clinical examination was repeated at 5 and 12 years after commencement of the study, resulting in repeat measurements of several key variables.

Whitehall II Study (WHII): (7) Whitehall II recruitment of 10,308 participants (70% men) between 1985 and 1988 involved 20 London based Civil service departments. Genetic samples were collected in 2004 from over 6,000 participants. The study is highly phenotyped for cardiovascular and other ageing related health outcomes, with 9 phases of follow up (5 with clinical assessment and biological sampling), over 20 years of follow up. A wide variety of health behaviour and 7 environmental data are also collected and the participants are consented for linkage to recorded clinical data such as Hospital Episode Statistics (HES), the Office of National Statistics mortality data and the national registry of acute coronary syndromes in England and Wales (Myocardial Ischaemia National Audit Project). (http://www.ucl.ac.uk/whitehallII/)

English Longitudinal Study of Ageing (ELSA): (8) This is a national cohort of participants (48% men) aged over 50 years recruited from the Health Surveys for England in 1998, 1999 and 2001. Genetic data were collected at Wave 2 of the study (2004/5). A wide range of phenotypic measures relevant to ageing are available. These measures were made at Wave 0 of the study (1998, 1999 and 2001) and at follow up (2004/5). Data on health behaviours and a wide range of health outcomes are available. Nearly all participants (97%) are also consented to linkage to routine data such as HES, which allows for the assessment of health outcomes and cause specific mortality. A case-control sample was selected using 412 cases and 1573 controls. Controls and cases were matched by sex and 5-year age bands at Wave 2. (http://www.ifs.org.uk/elsa/). Age in ELSA was collapsed at 90+ (and coded 90).

Caerphilly prospective study (CaPS): (9) This study is based on men aged between 45 to 59 years who resided in the small South Wales town of Caerphilly between the examination dates of 1979 &1983. Of the 2818 eligible, 2512, (89%) were recruited. The men were studied at baseline (Phase 1) and each subsequent 5 year period (Phase 2–5) and have therefore been followed up for around 20 years. An additional 447 patients were recruited at phase 2. The cohort has a wide range of cardiovascular phenotypes and at phase 3, cognitive function was also assessed, which has been supplemented with clinical dementia and cognitive impairment at phase 5. DNA was extracted from

blood samples collected in 1992–1994. Follow-up for disease outcomes is by self-report from participants, who are also linked to hospital episode discharge summaries for validation checks to comply with WHO criteria, as well as death certificates for fatal events.

References:

1. Shaper AG, Pocock SJ, Walker M, Cohen NM, Wale CJ, Thomson AG. British Regional Heart Study: cardiovascular risk factors in middle-aged men in 24 towns. Br Med J 1981;283:179-186,

2. Lawlor DA, Bedford C, Taylor M, Ebrahim S. Geographical variation in cardiovascular disease, risk factors, and their control in older women: British Women's Heart and Health Study. J Epidemiol Community Health 2003;57:134-140,

3. Wadsworth M, Kuh D, Richards M, Hardy R. Cohort Profile: The 1946 National Birth Cohort (MRC National Survey of Health and Development). Int J Epidemiol 2006;35:49-54,

4. Kuh D, Pierce M, Adams J, Deanfield J, Ekelund U, et al. Cohort profile: updating the cohort profile for the MRC National Survey of Health and Development: a new clinic-based data collection for ageing research. Int J Epidemiol 2011;40:e1-e9,

1. Shah T, Engmann J, Dale C, et al. Population genomics of cardiometabolic traits: design of the University College London-London School of Hygiene and Tropical Medicine-Edinburgh-Bristol (UCLEB) Consortium. PLoS One 2013;**8**(8):e71345.

6. Marmot MG, Smith GD, Stansfeld S, Patel C, et al. Health inequalities among British civil servants: the Whitehall II study. Lancet 1991;337:1387-1393,

7. Marmot MG, Banks J, Blundell R, Lessof C, and Nazroo J. Health, Wealth and Lifestyles of the Older Population in England: The 2002 English Longitudinal Study of Ageing. 2003;Institute for Fiscal Studies.

8. Bainton D, Miller NE, Bolton CH, Yarnell JW, et al. Plasma triglyceride and high density lipoprotein cholesterol as predictors of ischaemic heart disease in British men. The Caerphilly and Speedwell Collaborative Heart Disease Studies. Br Heart J 1992;68:60-66.

eTable 1. Details of published CARDIoGRAMplusC4D risk variants used in the risk analysis and their odds ratios, in their own data and for UCLEB data

RS number	CHR	Chr position (hg18)	Gene	risk allele/other	Risk allele freq.
CHD SNPs					
rs4845625	1	152688691	IL6R	T/C	0.47
rs602633	1	109623034	SORT1	C/A	0.77
rs11206510	1	55268627	PCSK9	T/C	0.84
rs17114036	1	56735409	PPAP2B	A/G	0.91
rs17464857	1	220829332	MIA3	T/G	0.87
rs1561198	2	85663500	VAMP5-VAMP8-GGCX	A/G	0.45
rs2252641	2	145517931	ZEB2-AC074093.1	G/A	0.46
rs6725887	2	203454130	WDR12	C/T	0.11
rs515135	2	21139562	APOB	G/A	0.83
rs6544713	2	43927385	ABCG5-ABCG8	T/C	0.3
rs9818870	3	139604812	MRAS	T/C	0.14
rs7692387	4	156854759	GUCY1A3	G/A	0.81
rs1878406	4	148613114	EDNRA	T/C	0.15
rs273909	5	131695252	SLC22A4-SLC22A5	C/T	0.14
rs9369640	6	13009427	PHACTR1	A/C	0.65
rs12190287	6	134256218	TCF21	C/G	0.59
rs2048327	6	160783522	SLC22A3-LPAL2-LPA	C/T	0.35
rs4252120	6	161063598	PLG	T/C	0.73
rs10947789	6	39282900	KCNK5	T/C	0.76
rs12205331	6	35006433	ANKS1A	C/T	0.81
rs3798220	6	160881127	SLC22A3-LPAL2-LPA	C/T	0.01
rs11556924	7	129450732	ZC3HC1	C/T	0.65
rs12539895	7	106879085	7q22	A/C	0.19

rs2023938	7	19003300	HDAC9	G/A	0.1
rs2954029	8	126560154	TRIB1	A/T	0.55
rs264	8	19857460	LPL	G/A	0.86
rs1333049	9	22115503	CDKN2BAS1	C/G	0.47
rs3217992	9	21993223	CDKN2BAS1	A/G	0.38
rs579459	9	135143989	ABO	C/T	0.21
rs2505083	10	30375128	KIAA1462	C/T	0.42
rs2047009	10	43859919	CXCL12	C/A	0.48
rs2246833	10	90995834	LIPA	T/C	0.38
rs11203042	10	90979089	LIPA	T/C	0.44
rs501120	10	44073873	CXCL12	A/G	0.83
rs12413409	10	104709086	CYP17A1-CNNM2-NT5C2	G/A	0.89
rs974819	11	103165777	PDGFD	A/G	0.29
rs9326246	11	116116943	ZNF259-APOA5-Apoa1	C/G	0.1
rs3184504	12	110368991	SH2B3	T/C	0.4
rs4773144	13	109758713	COL4A1-COL4A2	G/A	0.42
rs9319428	13	27871621	FLT1	A/G	0.32
rs9515203	13	109847624	COL4A1-COL4A2	T/C	0.74
rs2895811	14	99203695	HHIPL1	C/T	0.43
rs7173743	15	76928839	ADAMTS7	T/C	0.58
rs17514846	15	89217554	FURIN-FES	A/C	0.44
rs12936587	17	17484447	RAI1-PEMT-RASD1	G/A	0.59
rs15563	17	44360192	UBE2Z	C/T	0.52
rs2281727	17	2064695	SMG6	C/T	0.36
rs1122608	19	11024601	LDLR	G/T	0.76
rs2075650	19	50087459	ApoE-ApoC1	G/A	0.14
rs445925	19	50107480	ApoE-ApoC1	C/T	0.9
rs9982601	21	34520998	KCNE2	T/C	0.13

Stroke SNPS					
rs783396	6	107094063	AIM1	C/A	0.9
rs12425791	12	653745	NINJ2	A/G	0.19

eTable 2: Baseline characteristics by quintile of gene score

	Quintile of externally weighted gene score					
	1 (N=2371)	2 (N=2370)	3 (N=2370)	4 (N=2370)	5 (N=2370)	P value (trend)
Age	56.5 (5.4)	56.5 (5.4)	56.7 (5.4)	56.4 (5.4)	56.4 (5.4)	0.35
Sex (% male)	62.3(1478)	61.5 (1458)	64.6 (1531)	63.7 (1510)	63.8 (1511	0.11
% Smokers	54.4 (1290)	52.9 (1254)	52.5 (1244)	53.5(1268)	52.9 (1254)	0.46
Family history of CVD (%)	36.4 (863)	35.6 (844)	36.5 (865)	36.3 (860)	39.1 (927)	0.05
Townsend score	-0.03 (2.00)	0.13 (2.12)	-0.06 (1.86)	-0.001 (2.03)	-0.17 (1.91)	0.06
BMI (kg/m^2)	26.3 (4.0)	26.4 (4.1)	26.1 (3.9)	26.2 (4.0)	26.2 (4.1)	0.32
Total cholesterol (mmol/L)	6.28 (1.16)	6.28 (1.18)	6.28 (1.16)	6.39 (1.17)	6.45 (1.21)	$1.4x10^{-8}$
HDL cholesterol (mmol/L)	1.38 (0.42)	1.37 (0.41)	1.37 (0.41)	1.36 (0.41)	1.35 (0.40)	0.004
Systolic blood pressure (mmHg)	139.2 (23.6)	139.4 (23.5)	138.8 (23.1)	139.0 (23.9)	138.8 (22.9)	0.45
Diastolic blood pressure (mmHg)	82.1 (12.2)	82.0 (12.2)	82.0 (12.1)	82.3 (12.5)	82.1 (12.3)	0.72
Treated hypertension	12.4% (295)	13.4% (317)	12.5% (297)	12.2% (288)	11.9% (282)	0.28

eTable 3. AUROCs for QRISK score and combined score by study and overall

Study	N Cases/controls	QRISK score	QRISK+EW
BRHS	205/1933	0.659 (0.622-0.695)	0.647 (0.609-0.685)
BWHHS	268/1363	0.582 (0.544-0.619)	0.582 (0.545-0.619)
EAS	119/1002	0.659 (0.608-0.710)	0.630 (0.578-0.682)
MRC NSHD	235/397	0.618 (0.572-0.664)	0.600 (0.554-0.646)
WHII	142/1042	0.631 (0.585-0.678)	0.625 (0.577-0.673)
ELSA	109/2221	0.674 (0.626-0.722)	0.657 (0.605-0.709)
CAPS	366/2449	0.637 (0.605-0.668)	0.632 (0.600-0.664)
Combined studies (fixed effect)	1444/10407	0.635 (0.619-0.650)	0.623 (0.608-0.639)

eTable 4: Area under the receiver operating characteristic curve (AUROC; 95% CI) for participants aged under and over 60 years at baseline

	**AUROC (95% CI)**		
**Combined studies**	**Age<=60**<br>**N=8536**	**Age>60**<br>**N=3315**	**P value**
**GRS**	0.530 (0.509-0.551)	0.518 (0.492-0.544)	0.49
**QRISK**	0.648 (0.629-0.668)	0.604 (0.583-0.625)	0.004
**QRISK+EW**	0.638 (0.618-0.658)	0.592 (0.567-0.618)	0.006
Difference<br>(QRISK+EW)-QRISK<br>P value	-0.010 (-0.021 to 0.001)<br>P=0.09	-0.007 (-0.024 to 0.010)<br>P=0.40	0.77

eTable 5 : Net reclassification index based on addition of gene score to QRISK, calculated using 10% risk cut off, stratified by age group.

a) Age at baseline <=60 years

<=60 years	Number of people				
	QRISK + externally weighted gene score<br>NO CVD (N=9444.82+)		Reclassified		
Predicted risk<br>QRISK	<10	>=10	Increased risk	Decreased risk	Net correctly reclassified
<10<br>>=10	5276.3<br>784.50	700.01<br>2684.00	700.01	784.50	0.89%<br>(0.10 to 1.69)
Predicted risk<br>QRISK	QRISK + externally weighted gene score<br>CVD (N=889.26+)				
	<10	>=10			
<10<br>>=10	335.68<br>64.96	62.36<br>426.27	62.36	64.96	-0.29%<br>(-2.78 to 2.19)
NRI (95% CI)	0.60% (-2.01 to 3.21)<br>P=0.65				

b) Age at baseline >60 years

>60 years	Number of people				
	QRISK + externally weighted gene score<br>NO CVD (N=6483.8+)		Reclassified		
Predicted risk<br>QRISK	<10	>=10	Increased risk	Decreased risk	Net correctly reclassified
<10<br>>=10	198.72<br>371.56	85.54<br>5827.99	85.54	371.56	4.4%<br>(3.8 to 5.1)
Predicted risk<br>QRISK	QRISK + externally weighted gene score				

	CVD (N=808.55+)				
	<10	>=10			
<10<br>>=10	16.59<br>35.62	5.00<br>753.33	5.00	35.62	-3.8%<br>(-5.3 to -2.2)
NRI (95% CI)	0.6% (-1.0 to 2.3)<br>P=0.26				

+ Numbers inflated due to extra weighting assigned to three studies where samples of controls were taken

eTable 6: Net reclassification index based on addition of gene score to QRISK, calculated using 20% risk cut off, stratified by age group.

a) Age at baseline <=60 years

	Number of people				
<=60 years	QRISK + externally weighted gene score<br>NO CVD (N=9444.8+)		Reclassified		
Predicted risk<br>QRISK	<20	>=20	Increased risk	Decreased risk	Net correctly reclassified
<20<br>>=20	8329.5<br>191.20	450.93<br>473.19	450.93	191.20	-2.7%<br>(-3.3 to -2.2)
Predicted risk<br>QRISK	QRISK + externally weighted gene score<br>CVD (N=889.3+)				
	<20	>=20			
<20<br>>=20	655.63<br>45.60	79.14<br>108.89	79.14	45.60	3.8% (1.3 to 6.2)
NRI (95% CI)	1.0% (-1.5 to 3.5)<br>P=0.43				

b) Age at baseline >60 years

>60 years	Number of people				
	QRISK + externally weighted gene score<br>NO CVD (N=6483.8+)		Reclassified		
Predicted risk<br>QRISK	<20	>=20	Increased risk	Decreased risk	Net correctly reclassified
<20<br>>=20	1459.74<br>736.16	332.93<br>3955.00	332.93	736.16	6.2% (5.2 to 7.2)
Predicted risk<br>QRISK	QRISK + externally weighted gene score				

	CVD (N=808.55+)				
	<10	>=10			
<20<br>>=20	125.63<br>82.96	45.10<br>554.86	45.10	82.96	-4.7%<br>(-7.4 to -1.9)
NRI (95% CI)	1.5% (-1.4 to 4.5)<br>P=0.30				

+ Numbers inflated due to extra weighting assigned to three studies where samples of controls were taken
